# Discussion on “Testing small study effects in multivariate meta‐analysis” by Chuan Hong, Georgia Salanti, Sally Morton, Richard Riley, Haitao Chu, Stephen E. Kimmel and Yong Chen

**DOI:** 10.1111/biom.13345

**Published:** 2020-08-29

**Authors:** Hans C. van Houwelingen

**Affiliations:** ^1^ Department of Biomedical Data Sciences (retired) Leiden University Medical Center Leiden The Netherlands

## 1 INTRODUCTION

The paper under discussion (described as “the paper” in this discussion) introduces models for the effect of small studies in multivariate meta analysis. This is done under the working hypothesis that this can be analyzed by studying the effect of the study‐specific standard error on the outcomes of interest. That is fine with me. However, I am concerned about the way the data are modeled in the paper and the lack of transparency of the way the data are analyzed. In this discussion, I propose a modification of the model and a simplification of the analysis. I present a more detailed analysis of the two case studies. The *P*‐values obtained in that analysis are close to the ones obtained in the paper. Finally, a sharper correction for multiple testing is presented and the lack of interpretation of “just *P*‐values” is commented.

## 2 THE MODEL

### 2.1 Standardized effect size

The authors use the standardized effect size SND=Y/sd(Y), where *Y* is the outcome of interest and sd(Y) is its standard deviation. The concept comes from the social sciences and is used to correct for differences in scale between studies. Outcomes in different studies are all scaled to standarddeviation=1. The effect of an intervention is then given by the differences between the means of the treated group and untreated group. This is intrinsically different from the standardization used here. I would prefer not to apply the standardization and to use the more direct model of the outcome Y. The univariate models used in the paper are the meta‐regression models (Thompson and Higgins, 2002; Van Houwelingen *et al*., 2002).

(1) $$\text{Egger:}\;Y_{i}∼N\left(\alpha +\beta s_{i},s_{i}^{2}\right),$$

(2) $$\text{Lin-Chu:}\;Y_{i}∼N\left(\alpha +\beta \sqrt{\tau^{2}+s_{i}^{2}}),\tau^{2}+s_{i}^{2}\right).$$

Presenting the models this way does not really change them, but it helps to see them in the broader perspective of the meta‐regression models. If the relation between $\sqrt{\tau^{2}+s_{i}^{2}}$ and $s_{i}$ is approximately linear, the regression parts of the models are even approximately equivalent, but the variances are different if $\tau^{2}>0$. The confusing complication might be that the β in (2) pops up as *a* in Equation (4) of the paper. So, there is no discussion about what to test, but a difference of opinion about the way of presenting the data.

### 2.2 Comments on the models: alternative model

In his discussion of Lin and Chu (2018), Jackson (2018) showed dislike of the Lin‐Chu model . I share his concern. I want to add three points:

- If the model is meant to quantify the effect of selection, $s_{i}$ can be relevant but τ^2^ will not be known at the moment of selection and cannot act as a modifier of the study‐specific treatment effect. It can only be used retrospectively and not prospectively.
- If a parameter plays a role in both expected value and variance, the estimate of the expected value can be biased. (van Houwelingen, 1988)
- The Lin‐Chu model cannot be analyzed by GEE‐type methodology, unless the two τ^2^ 's are disconnected.

The drawback of the Egger model is that it does not contain a random effect term. A more suitable model would be a hybrid model

(3) $$\text{Hybrid}\;\mathrm{model:}\;Y_{i}∼N\left(\alpha +\beta s_{i},\tau^{2}+s_{i}^{2}\right).$$

This variance term in this model is flexible enough if there is a substantial random effect ($\tau^{2}>>0$), but it is quite rigid if $\tau^{2}=0$. The authors suggest a two‐step approach in which τ^2^ is estimated from the marginal model $Y_{i}∼N\left(\alpha ,\tau^{2}+s_{i}^{2}\right)$. This leads to the hybrid model $Y_{i}∼N\left(\alpha +\beta s_{i},{\hat{\tau }}^{2}+s_{i}^{2}\right)$. In this model, the variance is completely specified. I would suggest a more flexible variant:

(4) $$\text{Flexible}\;\text{Hybrid}\;\mathrm{model:}\;Y_{i}∼N\left(\alpha +\beta s_{i},\sigma^{2}\left({\hat{\tau }}^{2}+s_{i}^{2}\right)\right).$$

The advantage of this model over the Lin‐Chu model is that it can be fitted simply by standard models via the transformation

(5) $$\begin{array}{ccc} Y_{i}^{*} & = & Y_{i}/\sqrt{\left({\hat{\tau }}^{2}+s_{i}^{2}\right)}\;x_{0i}=1/\sqrt{\left({\hat{\tau }}^{2}+s_{i}^{2}\right)}\\ x_{1i} & = & s_{i}/\sqrt{\left({\hat{\tau }}^{2}+s_{i}^{2}\right)} \end{array}$$

The transformed model can be written as

(6) $$Y_{i}^{*}\;∼N\left(x_{i}\theta ,\sigma^{2}\right)\;\text{with}\;x_{i}=\left(x_{0i},x_{1i}\right)\;\text{and}\;\theta ={\left(\alpha ,\beta \right)}^{⊤}$$

It looks very much like the SND of the paper, but it is introduced for computational convenience only. The σ parameter is missing in the pseudo‐likelihood approach of the paper. Moreover model (6) gives more information on the data than the score test for β in the paper. Both α and β are estimated and the estimate does not depend on σ. This approach will be explored in Section 5 for the two case studies of the paper.

## 3 MULTIVARIATE DATA

The interest of the paper is in the situation of multiple outcome denoted by $Y_{1},\ldots ,Y_{J}$ with corresponding $\sigma_{1},\ldots ,\sigma_{J}$ and the development of a global test for the $\beta^{\prime }s$, that is a test for $\beta_{j}=0$ for j=1,…,J. As in the paper, we concentrate on J=2. The analysis suggested in Section 3 will yield estimates ${\hat{\theta }}_{1}$ and ${\hat{\theta }}_{2}$ with corresponding (estimated) covariate covariance matrices $C_{1}=\text{cov}\left({\hat{\theta }}_{1}\right)$ and $C_{2}=\text{cov}\left({\hat{\theta }}_{2}\right)$. The outcomes of interest need not be present for all studies. Lacking is the cross‐covariance matrix $C_{12}=\text{cov}\left({\hat{\theta }}_{1},{\hat{\theta }}_{2}\right)$. As in the GEE with a working independence model, that cross‐covariance matrix can be estimated from the set *A* of studies that have both outcomes, as

(7) $$\begin{array}{ccc} C_{12} & = & {\left(x_{1}^{⊤}x_{1}\right)}^{-1}\sum_{i\in A}\left(x_{1i}^{⊤}\left(Y_{1i}^{*}-{\hat{Y}}_{1i}^{*}\right)\left(Y_{2i}^{*}-{\hat{Y}}_{2i}^{*}\right)x_{2i}\right)\\ & & \times \;{\left(x_{2}^{⊤}x_{2}\right)}^{-1}. \end{array}$$

Here $x_{1i}$ and $x_{2i}$ are the *i*the row‐vectors of the x‐matrices $x_{1}$ and $x_{2}$ for outcomes *Y*_1_ and *Y*_2_, respectively, as defined in (5) and (6). Furthermore, $\hat{Y_{1}^{*}}=x_{1}{\hat{\theta }}_{1}$ and $\hat{Y_{2}^{*}}=x_{2}{\hat{\theta }}_{2}$. The estimate depends on the covariance of $Y_{1}^{*}$ and $Y_{2}^{*}$ and the amount of overlap of the two outcomes. From the large covariance matrix $C_{\theta }=\text{cov}\left(\hat{\theta }\right)$, $C_{\beta }=\text{cov}\left({\hat{\beta }}_{1},{\hat{\beta }}_{2}\right)$ can be extracted and from that a bivariate χ^2^‐test for $H_{0}:\beta_{1}=\beta_{2}=0$ ($\chi_{\left[2\right]}^{2}={\hat{\beta }}^{⊤}C_{\beta }^{-1}\hat{\beta }$).

If there is little overlap, the test boils down to adding the two univariate $\chi_{\left[1\right]}^{2}$'s. This procedure must have some relation with the one in the paper, but I found that quite hard to read. The supplementary material was not very helpful. It is even not clear how outcomes are handled that are present in all studies.

The approach suggested here can be extended to J>2, but the large covariance matrix for $\hat{\beta }$ might fail to be nonnegative definite.

## 4 CASE STUDIES REVISITED

### 4.1 Case study 1: Heart failure

The two outcomes considered in the paper are *Y*_1_ = “all‐cause mortality” and *Y*_2_= “Mental quality of Life.” The related standard errors are denoted by *s*_1_ and *s*_2_. The data are shown in Figure 1 of the paper. The paper only reports the *P*‐values of the univariate tests: *P* = 0.06 for *Y*_1_ and *P* = 0.09 for *Y*_2_ and the multivariate *P* = 0.03. (Unfortunately the *P*‐values are presented in the wrong order, which caused serious confusion). The data were analyzed with model (4) using the transformation of (5) for ease of computation.

Table 1 shows the basic information of mean, standard deviation, and correlation. The most striking feature is the high correlation between *s*_1_ and *s*_2_.

**TABLE 1:** Basic data for case study 1: *n*, mean, standard deviation, and correlations

	*n*	Mean	SD	*Y*_1_	*s*_1_	*Y*_2_	*s*_2_
*Y*_1_	34	−0.32	0.60	1			
*s*_1_		0.51	0.49	−0.154	1		
*Y*_2_	12	0.18	0.24	0.034	0.608	1	
*s*_2_		0.17	0.11	−0.168	0.874	0.445	1

Table 2 shows the results of my analysis sketched above.

**TABLE 2:** Regression of *Y* on *s* for case study 1. p* is the *P*‐value if σ is fixed at σ=1

	τ^2^	$\hat{\alpha }$ (se)	$\hat{\beta }$ (se)	σ	χ^2^	df	*P*	p∗
*Y*_1_	0	−0.04 (0.09)	−0.62 (0.30)	0.91	4.04	1	0.037	0.058
*Y*_2_	0.17	−0.07 (0.13)	1.51 (0.91)	1.02	2.72	1	0.099	0.093
both	(covariance of the $\hat{\beta }$'s equals −0.0017)				7.04	2	0.030	

The first observation is that despite the different approaches (score test vs Wald‐test) the *P*‐values for σ=1 agree with the ones given in the paper. Moreover the *P*‐value for the multivariate test agrees with the one given in the paper. Finally, it also shows the potential of the multivariate approach. The multivariate $\chi_{\left[2\right]}^{2}$ is larger than the sum of the two $\chi_{\left[1\right]}^{2}$'s.

### 4.2 Case study 2: Neuroblastoma

The data are shown in Figure 1 of the paper. As there are much more observations for Disease‐Free Survival (DFS) than for Overall Survival (OS), DFS is taken as *Y*_1_ and overall OS as *Y*_2_. Table 3 shows again a very high correlation between *s*_1_ and *s*_2_ and a bit confusing pattern of correlations. One should realize that there are only 10 studies with OS outcome and only eight studies with both DFS and OLS and all correlations involving *Y*_2_ or *s*_2_ are very shaky.

**TABLE 3:** Basic data for case study 2: *n*, mean, standard deviation, and correlations

	*n*	Mean	SD	*Y*_1_	*s*_1_	*Y*_2_	*s*_2_
*Y*_1_	50	1.85	1.14	1			
*s*_1_		0.70	0.29	0.475	1		
*Y*_2_	10	1.36	0.97	0.639	−0.614	1	
*s*_2_		0.63	0.31	−0.451	0.972	0.483	1

The results in Table 4 are quite similar to those in Table 2. The difference is that the $\chi_{\left[2\right]}^{2}$ is smaller than the sum of the two $\chi_{\left[1\right]}^{2}$'s. The role of global testing is discussed in the next section.

**TABLE 4:** Regression of *Y* on *s* for case study 1. p* is the *P*‐value if σ is fixed at σ=1

	τ^2^	$\hat{\alpha }$ (se)	$\hat{\beta }$ (se)	σ	χ^2^	df	*P*	p∗
*Y*_1_	0.72	0.89 (0.34)	1.34 (0.51)	0.85	6.89	1	0.009	0.025
*Y*_2_	0.45	0.78 (0.87)	0.87 (1.43)	0.96	0.37	1	0.544	0.562
both	(covariance of the $\hat{\beta }$'s equals 0.051)				7.07	2	0.029	

The high correlations between *s*_1_ and *s*_2_ in both examples suggest a common measure like sample size that could be used for all outcomes.

## 5 MULTIPLE TESTING

Some clarification of the role of global (multivariate) testing could be helpful. The question is what could be said about the univariate tests if the multivariate test is significant at α=0.10. There is some interesting theory in Shaffer (1986) and Goeman and Solari (2011). It gives the following improvement over Bonferoni‐Holmes:

*Let the ordered p‐values of the J outcomes be* $p_{\left(1\right)},p_{\left(2\right)},\ldots ,p_{\left(J\right)}$.

*The corresponding univariate nulhypotheses can be rejected sequentially as long as* $p\left(j\right)\leq \alpha /t_{j}$ *with* t=(J−1,J−1,J−2,…,1).

In the examples with J=2, the $t_{j}$'s are given by t=(1,1). This implies that the nulhypotheses in the examples can be rejected at α=0.10 if the multivariate test is significant.

Actually, I think there is too much emphasis in the paper on *P*‐values. That is the weakness of score tests: no effect measures are given, only *P*‐values. I strongly suggest to give estimates of the β parameters and some help in interpreting them.
